# Evaluation of Biopesticides for Management of *Bemisia tabaci* Middle East-Asia Minor 1 (Hemiptera: Aleyrodidae) in Florida

**DOI:** 10.3390/insects15060438

**Published:** 2024-06-10

**Authors:** Marcelo Dimase, Sriyanka Lahiri, Julien Beuzelin, Sam Hutton, Hugh Adam Smith

**Affiliations:** 1 Gulf Coast Research and Education Center, Department of Entomology and Nematology, University of Florida, Wimauma, FL 33598, USA; lahiris@ufl.edu (S.L.); hughasmith@ufl.edu (H.A.S.); 2 Everglades Research and Education Center, Department of Entomology and Nematology, University of Florida, Belle Glade, FL 33430, USA; jbeuzelin@ufl.edu; 3 Gulf Coast Research and Education Center, Department of Horticultural Sciences, University of Florida, Wimauma, FL 33598, USA; sfhutton@ufl.edu

**Keywords:** *Bemisia tabaci*, tomato, integrated pest management (IPM), insecticide resistance, biopesticides, *Beauveria bassiana*, *Cordyceps javanica*, agricultural sustainability

## Abstract

**Simple Summary:**

In agriculture, managing pests like the sweetpotato whitefly, *Bemisia tabaci* MEAM1, which can severely affect the development and yield of vegetable crops, is crucial. This study explored different insecticide rotations to control this pest on tomato plants. We compared standard synthetic insecticide rotations with biopesticide rotations including biochemical and microbial products alone or combined. Our trials, conducted in the spring and fall of 2023, examined how different insecticide rotations impacted the number of whiteflies. The results showed that while traditional synthetic insecticides consistently reduced the whitefly numbers, biopesticides also provided whitefly control to a lower extent. Overall, although a standard synthetic insecticide rotation adopted in Florida to manage *B. tabaci* MEAM1 was very effective, microbial biopesticides could become an option to reduce the use of synthetic insecticides and potentially mitigate the risk resistance development. These findings offer farmers new options to manage whiteflies effectively while also considering environmental sustainability. In summary, this research contributes to the ongoing efforts in agriculture to balance effective pest control with the need to protect the environment and reduce chemical usage.

**Abstract:**

The sweetpotato whitefly, *Bemisia tabaci* MEAM1, is a pest known to significantly impact tomato development and yields through direct damage and virus transmission. To manage this pest, the current study compared the effectiveness of various insecticide rotations. Field trials included rotations involving synthetic insecticides, biochemicals, and microbial agents, applied according to their highest labeled concentrations. The results indicated that while standard synthetic insecticides consistently reduced whitefly egg and nymph counts significantly, microbial biopesticide rotations also achieved reductions, although less consistently. This study demonstrated that while traditional chemical treatments remain highly effective, microbial biopesticides containing *Beauveria bassiana* and *Cordyceps javanica* present a viable alternative to manage MEAM1 in tomato fields. The data generated in this study provided baseline information for further investigations to determine the potential for optimizing integrated pest management (IPM) and insecticide resistance management (IRM) strategies by incorporating microbial biopesticides in rotations with a variety of modes of action to sustainably manage *B. tabaci* MEAM1 populations in agricultural settings.

## 1. Introduction

The sweetpotato whitefly, *Bemisia tabaci* (Gennadius) (Hemiptera: Aleyrodidae), represents an important threat to a diverse range of vegetable crops, including tomato (*Solanum lycopersicum* L.) (Solanaceae) [1]. *Bemisia tabaci* is a complex of multiple cryptic species, differentiated by the mitochondrial cytochrome c oxidase subunit 1 (COI) DNA sequence [2,3]. Despite their identical appearance, these cryptic species exhibit genetic diversity associated with distinct behavior in terms of host preference, the ability to transmit viruses, and insecticide susceptibility [2,3,4,5,6,7]. The *B. tabaci* complex includes the Middle East-Asia Minor 1 (MEAM1) and Mediterranean (MED) species, with MEAM1 recognized as a prominent pest in Florida tomato [1,2,3,8].

The transmission of tomato yellow leaf curl virus (TYLCV) by MEAM1 represents a serious challenge for tomato yield and quality [9]. Additionally, the feeding activity of nymphs can lead to disorders such as squash silverleafing [10] and tomato irregular ripening [11]. In agricultural settings, the use of insecticides has been the primary defense against MEAM1 damage in Florida [8,12,13,14,15]. However, repetitive application of the same mode of action may induce insecticide resistance in pest populations [16]. Insecticide resistance occurs when an insecticide consistently fails to provide pest control, due to heritable alterations in the susceptibility of a pest population when used as specified on the product label [17].

Many groups of conventional insecticides with distinct modes of action are routinely used to manage MEAM1 in Florida, including dinotefuran, cyantraniliprole, buprofezin, afidopyropen, and pyriproxyfen. The neonicotinoid dinotefuran, whose mode of action (MoA) was categorized as 4A by the Insecticide Resistance Action Committee (IRAC), is an acetylcholine receptor agonist that has systemic activity [17]. Group 4 insecticides are optimally used at-transplanting and 3–5 weeks after transplanting [18]. Dinotefuran is frequently used at planting, via drip irrigation, and through foliar application [14,19]. This insecticide became commercially available in Florida in 2005 [19], and it is known for its high efficacy against MEAM1 [15,20].

Cyantraniliprole (MoA 28) is a ryanodine receptor agonist that has systemic activity, which impacts whiteflies by interfering with their calcium signaling pathways [21]. This insecticide offers a broader spectrum of pest control than group 4 insecticides; thus, it should be applied at later crop stages when caterpillars and leafminers may cause economic damage [18]. In Florida, cyantraniliprole became commercially available in 2014; however, low to moderate resistance to this insecticide has been reported in Florida and Georgia in less than a decade [22,23].

Buprofezin (MoA 16) and pyriproxyfen (MoA 7C) are Insect Growth Regulators (IGRs) that have been highly effective against egg and nymphal stages of MEAM1 populations in Florida tomato [8]. Buprofezin is a selective insecticide for the management of certain hemipterans such as whiteflies, which interferes with chitin biosynthesis and cuticle formation through contact [24]. Pyriproxyfen is a juvenile hormone mimic (JHM) that affects hormonal balance in insects, suppressing embryogenesis, metamorphosis, and adult formation via translaminar activity [25]. MEAM1 populations have developed resistance to pyriproxyfen worldwide [26,27]. While there are no reports of MEAM1 resistance to buprofezin in Florida, other *B. tabaci* species have developed resistance to this insecticide in other regions [28,29,30,31,32].

Afidopyropen (MoA 9D) provides an additional MoA by targeting the chordotonal organs of whiteflies, interfering with insect feeding and movement [33]. Afidopyropen is primarily translaminar, with limited systemic activity [33,34]. This insecticide became commercially available in Florida in 2018; nonetheless, different species within the *B. tabaci* complex exhibited some degree of resistance to afidopyropen in Florida [23] and across the globe [35,36]. Given the global trend of insecticide resistance, alternative integrated pest management (IPM) and insecticide resistance management (IRM) strategies are necessary to maintain the efficacy of synthetic insecticides, while providing sustainable pest management.

In recent years, there has been a growing interest in the use of biopesticides as a part of IPM programs. Biopesticides, including biochemical and microbial insecticides, offer a more environmentally friendly approach, potentially reducing the reliance on synthetic chemicals and mitigating resistance development. Biochemical insecticides, such as insecticidal soaps and mineral oils, have shown potential in managing *B. tabaci* populations by mechanisms of action that likely do not select for resistance, including suffocation and anti-feedant properties [37,38,39,40,41,42]. Similarly, microbial insecticides containing *Beauveria bassiana* (Balsamo) Vuillemin (Hypocreales: Cordycipitaceae) and *Cordyceps javanica* (Kobayasi & Shimizu) (Hypocreales: Cordycipitaceae) as active ingredients have demonstrated efficacy against *B. tabaci* through the pathogenic activity of the fungi [41,43,44,45]. Despite the potential benefits of biopesticides, their performance can be variable and influenced by environmental conditions such as UV light, as well as application methods [43,46,47,48]. Therefore, a thorough understanding of their effectiveness, particularly in rotation with synthetic insecticides, is crucial for their successful integration into IPM and IRM programs.

The potential of biopesticides to mitigate resistance development is economically desirable, whereas the potential to reduce the negative impacts of synthetic insecticides to pollinators, applicators, and the environment is socially desirable. Therefore, the goal of the present study was to address the gaps in understanding how biopesticide rotations compare with standard synthetic insecticide rotations in managing *B. tabaci* and TYLCV. We hypothesized that a strategic rotation of biopesticides and synthetic insecticides will equally provide effective control of *B. tabaci*. Our specific objective was to evaluate the efficacy of distinct biopesticide rotations compared with a standard rotation of synthetic insecticides in managing MEAM1. The current study aims to provide insights into the optimization of pest management strategies that balance efficacy with sustainability, contributing to the broader goals of IPM and IRM in agriculture.

## 2. Materials and Methods

### 2.1. Treatments

Field experiments were conducted in the spring and fall of 2023 at the University of Florida (UF) Gulf Coast Research and Education Center (GCREC) to evaluate the efficacy of distinct biopesticide rotations compared with a standard rotation of synthetic insecticides to control MEAM1. The treatments are detailed in Table 1. The first treatment, serving as an untreated control, involved no material application. In subsequent treatments, the highest concentration of each insecticide according to each product label was used. The second treatment consisted of a rotation of biochemical insecticides applied once a week between the third and eighth week after transplanting tomato to the field. This rotation included the insecticidal soap M-Pede (Gowan Company, Yuma, AZ, USA), the mineral oils SuffOil-X (hereafter SX) (BioWorks^®^, Victor, NY, USA), and Trilogy^®^ (Certis Biologicals, Columbia, MD, USA). M-Pede was applied in the third and fourth weeks post transplanting, SuffOil-X in the fifth and sixth weeks, and Trilogy^®^ in the seventh and eighth weeks. The third treatment comprised a rotation of two microbial insecticides applied weekly from the third to the eighth week post transplanting. These included BotaniGardES (hereafter BG) (Certis Biologicals, Columbia, MD, USA), which contains *B. bassiana* as the active ingredient, and PFR-97 20WDG (hereafter PFR) (Certis Biologicals, Columbia, MD, USA), which contains *C. javanica* (formerly *Isaria fumosorosea*) as the active ingredient. BG was used in the third, fourth, and fifth week post transplanting, whereas PFR was used in the sixth, seventh, and eighth week after transplanting. The fourth treatment consisted of combinations of biochemical and microbial insecticides. In this regimen, BG + M-Pede were applied in the third week, BG + SX in the fourth week, BG + Trilogy in the fifth week, PFR + M-Pede in the sixth week, PFR + SX in the seventh week, and PFR + Trilogy in the eighth week. The fifth treatment, which was a standard rotation of synthetic insecticides, started with a dinotefuran (Venom^®^, Valent USA, Walnut Creek, CA, USA) drench application at planting, followed by applications of various insecticides with different modes of action. Dinotefuran was applied again three weeks after transplanting tomato seedlings to the field. Cyantraniliprole (Exirel^®^, FMC Corporation, Philadelphia, PA, USA) was applied in the fifth week, buprofezin (Courier^®^ 70WP, Nichino America, Inc., Wilmington, DE, USA) in the sixth week, afidopyropen (Sefina^®^, BASF, Research Triangle Park, NC, USA) in the seventh week, and pyriproxifen (Knack^®^, Valent USA, Walnut Creek, CA, USA) in the eighth week after transplanting.

### 2.2. 2023 Spring Trial

The spring field trial was organized into four replicates, with each comprising five treatments in a randomized complete block design. This setup consisted of 20 plots placed in single rows, with each plot accommodating 14 plants spaced ~45 cm apart. Overall, the trial included 280 plants. The experiment was conducted using hybrid tomato seeds of the Florida 91 variety (Seedway LLC, Hall, NY, USA). Planting began on 16 February 2023, with tomato seeds sown in five seedling trays, with each containing 128 cells, resulting in 640 seeds. Seedlings were fertilized once with 1.5 g of Osmocote^®^ Plus 15-9-12 fertilizer (The Scotts Company, Marysville, OH, USA) and watered every 2–3 days. To obtain uniform plants for transplantation, the plant growth regulator Dazide^®^ 85 WSG (Fine America, Inc., Walnut Creek, CA, USA) was applied at a rate of 8 g per gallon after three weeks of sowing, with leaves being sprayed until dripping wet. Transplantation of the best 280 seedlings into the field occurred on 27 March 2023. Tomato transplants were established in 20 cm high, 80 cm wide beds of Myakka fine sand, spaced on 1.5 m centers, and covered with white impermeable plastic mulch (Kennco Manufacturing, Ruskin, FL, USA). After transplanting, field plots consisted of 14 plants in a 6.4 m long row that was separated by 3 m of unplanted beds within rows. Then, seedlings were watered and a dinotefuran drench was applied to treatment 5. The subsequent treatments, including foliar applications and drenches, were applied to different treatment groups as described above. Foliar applications were performed with a CO_2_-pressurized backpack sprayer, fitted with Albuz orange nozzles (Evreux, France), pressurized to 241.3 kPa (35 psi), and calibrated to deliver 560 to 840 L/ha (60–90 gallons/acre), depending on the height of the crop. Sampling began with a pre-sample collection three weeks after transplanting tomato seedlings on 17 April 2023, and ended with a fifth sample collected on 22 May 2023.

### 2.3. 2023 Fall Trial

The fall trial was expanded to include three-row plots. This setup involved four replicates arranged in a randomized complete block design, each with five treatments spread across 20 plots. Each row hosted 14 plants, maintaining a 45 cm spacing. The entire trial required 840 plants. For the fall trial, the same seed source and agronomic practices were implemented as described in the spring trial. Seeding for the fall trial began on 8 August 2023, with the seeds planted in ten seedling trays (128 cells each), totaling 1280 seeds. The best 840 seedlings were transplanted into the field on 11 September 2023. A dinotefuran drench was applied to treatment 5 at planting immediately after plants were watered. The subsequent treatments involved various sprays and drenches as described above, with changes in the application methods as the trial progressed. The major change in the application method in the fall trial included the adoption of an air-boom sprayer to improve plant coverage, beginning with the treatments applied at the sixth week after transplanting, when plants were tall enough and tied. The sprayer was set to deliver a similar concentration of insecticides as described in the spring trial. In the fall trial, a pre-sample collection was conducted three weeks after transplanting tomato seedlings on 2 October 2023 and ended with a sixth sample collected on 13 November 2023.

### 2.4. Data Collection

#### 2.4.1. Weather

The weather data, including daily temperature, humidity, and cumulative rainfall, were recorded from the Florida Automated Weather Network (FAWN) database during the period of both spring and fall trials.

#### 2.4.2. Whitefly Eggs and Nymphs

Whitefly eggs and nymphs originating from natural infestations were evaluated by examining the bottom leaflet of the sixth leaf from the tomato apex of ten central plants in each plot [49]. In the lab, ten leaflets per plot, one from each plant, were analyzed using a stereomicroscope, focusing on the underside of the leaflets. Starting from the third week post transplanting, the presence of eggs and nymphs in different developmental stages (first and middle stages—second, third, and fourth instars) was recorded for each trial once a week for a period of five and six weeks during the spring and fall of 2023, respectively.

#### 2.4.3. Statistical Analysis

All datasets underwent statistical analysis using the R statistical software version 4.3.1 [50]. Analysis of variance (ANOVA) assumptions were checked using the “car” package [51]. Residual plots and a Shapiro–Wilk test were employed to assess the normality of residuals, whereas Levene’s test was used to evaluate the homogeneity of variances. The main effects of insecticide rotation, sampling dates, and their interaction on counts of whitefly eggs and nymphs were assessed using linear mixed-effect models implemented with the “lme4” package [52]. These models were fitted to both log-transformed egg and nymph counts. The model per sample included the fixed effects of sample date, insecticide rotation, and their interaction, while replication was a random effect. The combined model included the fixed effect of insecticide rotation, while replication and sample date were random effects. The estimated marginal egg/nymph means for treatments within each sample and total samples combined were then calculated using the “emmeans” function from the “emmeans” package [53]. Pairwise comparisons for mean eggs and nymphs, both per sample and total combined, were performed with the “TukeyHSD” function using the “stats” package [50]. All results presented in tables and figures are displayed as untransformed data.

## 3. Results

### 3.1. Weather

Throughout the spring of 2023, the mean daily temperature and relative humidity were recorded at 24.1 °C (20.0 °C min, 27.0 °C max) and 73.4% (55% min, 82% max), respectively, observed from 27 March to 22 May 2023 [54]. During the fall of 2023, the mean daily temperature and relative humidity were recorded at 24.0 °C (15.9 °C min, 28.5 °C max) and 78.3% (53% min, 87% max), respectively, observed from 11 September to 13 November 2023 [54]. Furthermore, cumulative rainfall data for these intervals provided insights into moisture conditions during our trials. For spring 2023, cumulative rainfall was 8.7 cm. One heavy and one moderate rainfall occurred during the 2023 spring trial on 17 April 2023 (5.0 cm) and 24 April 2023 (2.1 cm), respectively. The second rainfall event during the spring of 2023 occurred on the day after we collected our first sample, which was unlikely to impact our data. Cumulative rainfall was 20.4 cm during the 2023 fall trial. One heavy and one moderate rainfall occurred during the 2023 fall trial on 29 September 2023 (8.0 cm) and 30 September 2023 (3.6 cm), respectively [54]. Those events took place ~one week before we collected data for our first sample, which was unlikely to affect our data during the fall of 2023. Throughout our field trials, the prevailing weather conditions closely aligned with the optimal temperature range (16–24 °C) conducive to *B. tabaci* development in south Florida [55].

### 3.2. Rotation Effects on Whitefly Eggs and Nymphs

In the field trials conducted at the GCREC during the spring and fall of 2023, the impacts of rotation treatment, sample date, and their interaction on *B. tabaci* eggs and nymphs were assessed. The statistical analysis revealed significant main effects of rotation treatment and sample date on both eggs and nymphs, with variations observed between both seasons as shown in Table 2. In the spring of 2023, rotation treatment exhibited a significant effect on both eggs (F_4,75_ = 10.11, *p* < 0.0001) and nymphs (F_4,75_ = 21.63, *p* < 0.0001). Similarly, sample date demonstrated a significant effect on eggs (F_4,75_ = 16.44, *p* < 0.0001) and nymphs (F_4,75_ = 24.94, *p* < 0.0001) during the same season. However, the interaction between rotation treatment and sample date did not exhibit a significant effect on either egg (F_16,75_ = 1.32, *p* = 0.2099) or nymphs (F_16,75_ = 1.33, *p* = 0.2022) in the spring trials. During the fall trials of 2023, rotation treatment significantly influenced eggs (F_4,87_ = 4.49, *p* = 0.0024) and nymphs (F_4,87_ = 21.47, *p* < 0.0001). In contrast, while sample date had no significant impact on egg counts (F_5,87_ = 0.54, *p* = 0.7487), it significantly impacted nymph counts (F_5,87_ = 9.04, *p* < 0.0001) during the fall trials. The interaction between rotation treatment and sample date did not have a significant effect on either egg (F_20,87_ = 0.96, *p* = 0.5151) or nymphs (F_20,87_ = 1.31, *p* = 0.1831) during the fall trials.

### 3.3. 2023 Spring Trial

**Figure 1:**
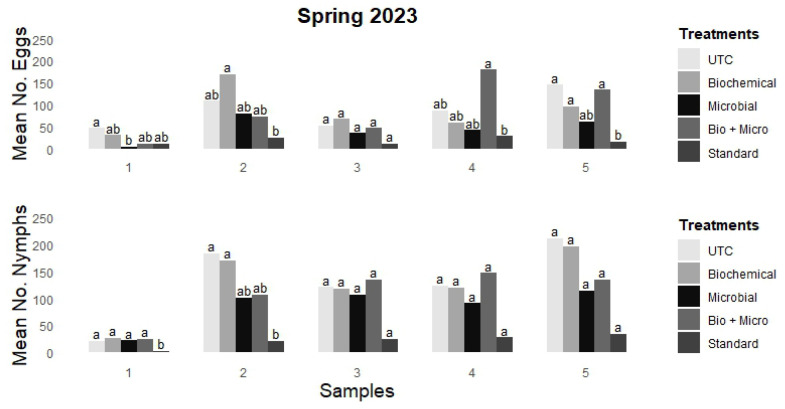
Mean number of eggs (**top**) and nymphs (**bottom**) per sample for field trial carried out at GCREC during spring 2023. Tukey’s mean separation letter designate statistical differences among treatments within sample. Columns with different letters in each sample are statistically different (*p* < 0.05).

The rotation responses are represented in Figure 1 as mean egg and mean nymph counts across five samples. The untreated control (UTC) had the highest mean counts overall, with egg counts ranging from 47.2 to 145.8 (samples 1 and 5, respectively) and nymph counts from 21.8 to 211.4 (samples 1 and 5, respectively). The biochemical rotation followed, with egg counts varying from 31.0 (M-Pede) to 169.5 (M-Pede) (samples 1 and 2, respectively) and nymph counts from 26.5 (M-Pede) to 195.6 (Trilogy) (samples 1 and 5, respectively). The biochemical plus microbial (bio + micro) rotation showed egg counts from 11.2 (BG + M-Pede) to 179.5 (PFR + M-Pede) (samples 1 and 4, respectively) and nymph counts from 22.2 (BG + M-Pede) to 146.7 (PFR + M-Pede) (samples 1 and 4, respectively). There were no significant differences among these three rotations across all samples for both eggs and nymphs. In contrast, the standard synthetic rotation consistently and significantly reduced both egg and nymph counts, with eggs varying from 11.9 (cyantraniliprole) to 28.6 (buprofezin) (samples 3 and 4, respectively) and nymphs from 2.25 (dinotefuran) to 33.6 (afidopyropen) (samples 1 and 5, respectively). For sample 3, the standard rotation impact on egg and nymph counts with cyantraniliprole was marginal and not statistically significant (11.9 and 24.6, respectively) compared to the UTC (53.3 and 120.8, respectively). For samples 4 and 5, the standard rotation also exhibited a marginal reduction in nymph counts (27.6 with buprofezin and 33.6 with afidopyropen, respectively), which was not significantly different from the UTC (123.7 and 211.4, respectively). Notably, the microbial rotation marginally reduced the number of eggs, with only one statistically significant reduction (4.5 with BG observed in sample 1 compared to the UTC (47.2)). Furthermore, the influence of microbial rotations on nymph counts was comparable to the UTC across all samples, with reductions not reaching statistical significance. Nevertheless, marginal reductions in nymph counts were observed across all samples, ranging from 91.1 in sample 4 with PFR to 114.8 in sample 5 with PFR compared to the UTC (123.7 and 211.4, respectively).

**Figure 2:**
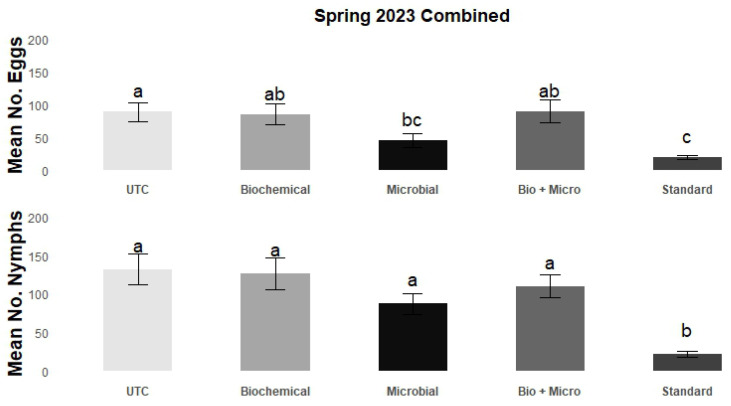
Mean (±SEM) number of eggs (**top**) and nymphs (**bottom**) combined for field trial carried out at GCREC during spring 2023. Tukey’s mean separation letter designates statistical differences among treatments (insecticide rotations). Columns with different letters are statistically different (*p* < 0.05).

These findings are consolidated in Figure 2, which illustrates the combined impact of the studied rotations over the entire spring trial period. The standard rotation demonstrated clear superiority in its efficacy when compared to the other rotations, significantly reducing nymph counts to an average of 21.8 ± 3.9, which represents an 84.3% reduction compared to the UTC. While its impact on mean egg counts was not significantly different from the microbial rotation (44.9 ± 10.2), it significantly outperformed the other rotations, reducing mean egg counts to 18.9 ± 3.6, representing a 78.7% reduction compared to the UTC. Similarly to the results by sample, no significant differences in both egg and nymph counts were observed among the UTC (88.9 ± 14.0 and 132.1 ± 20.2, respectively), biochemical (84.6 ± 16.0 and 126.0 ± 20.6, respectively), and bio + micro rotations (88.3 ± 17.5 and 109.8 ± 14.9, respectively) in the combined results. By contrast, the microbial rotation delivered an overall significant reduction of 49.5% in mean egg counts (44.9 ± 10.2) when compared to the UTC (88.9 ± 14.0), but this reduction was not significantly different compared to the biochemical (84.6 ± 16.0) and bio + micro rotations (88.3 ± 17.5). Although the microbial rotation provided a notable reduction of 31.1% in mean nymph counts (87.2 ± 13.5) in relation to the UTC, this was not statistically significant compared to UTC (132.1 ± 20.2), biochemical (126.0 ± 20.6), or bio + micro rotation (109.8 ± 14.9).

### 3.4. 2023 Fall Trial

**Figure 3:**
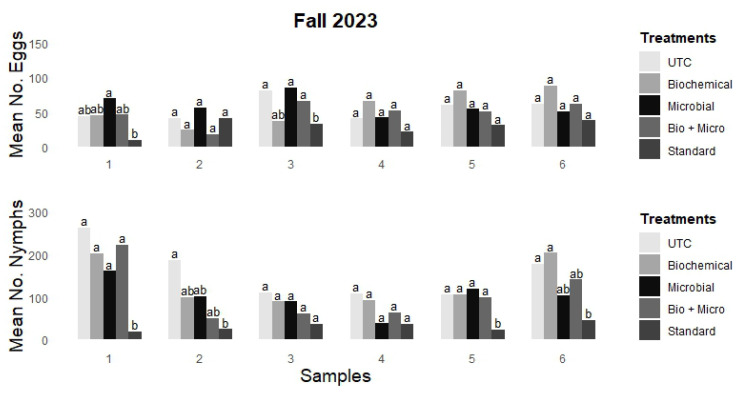
Mean number of eggs (**top**) and nymphs (**bottom**) per sample for field trial carried out at GCREC during fall 2023. Tukey’s mean separation letter designates statistical differences among treatments within sample. Columns with different letters in each sample are statistically different (*p* < 0.05).

Both mean egg and nymph counts by rotation were recorded across six samples during the fall 2023 field trial, as shown in Figure 3. There were no significant differences in egg counts between the untreated control (UTC) and the other treatments, except when compared with the standard rotation in most samples. The UTC exhibited mean egg counts from 40.4 to 81.5 (samples 4 and 3, respectively) and nymph counts ranging from 106.1 to 262.8 (samples 5 and 1, respectively). The biochemical rotation had mean egg counts varying from 24.2 (M-pede) to 87.9 (Trilogy) (samples 2 and 6, respectively) and mean nymph counts ranging from 88.9 (SX) to 202.9 (Trilogy) (samples 3 and 6, respectively). The microbial rotation showed mean egg counts from 41.8 (PFR) to 85.4 (BG) (samples 4 and 3, respectively) and nymph counts from 37.7 (PFR) to 160.5 (BG) (samples 4 and 1, respectively). The bio + micro treatment displayed mean egg counts from 18.0 (BG + SX) to 66.2 (BG + Trilogy) (samples 2 and 3, respectively) and nymph counts from 49.2 (BG + SX) to 222.5 (BG + M-Pede) (samples 2 and 1, respectively). In contrast, the standard synthetic rotation significantly reduced mean counts in most of the analyzed samples, with eggs counts varying from 10.2 (dinotefuran) to 41.0 (dinotefuran) (samples 1 and 2, respectively) and nymph counts varying from 19.5 (dinotefuran) to 45.0 (pyriproxifen) (samples 1 and 6, respectively). Notwithstanding, this pattern showed slightly more consistency in the decrease in nymphs counts. The standard rotation did not significantly reduce the number of nymphs in samples 3 (cyantraniliprole) and 4 (buprofezin) (36.9 and 36.4, respectively), when compared to the UTC (110.4 and 108.6, respectively). However, this ~3-fold marginal difference was consistent and notable. On the other hand, the standard rotation exhibited slightly less consistency in the decrease in egg counts. The standard rotation did not significantly reduce the eggs counts in samples 2 (dinotefuran), 4 (buprofezin), 5 (afidopyropen), and 6 (pyriproxifen) (41.0, 21.4, 31.1, and 37.9, respectively) in comparison to the UTC (41.0, 40.4, 60.6, and 62.1, respectively). Notably, the standard rotation provided an approximate 2-fold marginal reduction in egg counts compared to the UTC, except in the second sample, where egg counts were equal.

**Figure 4:**
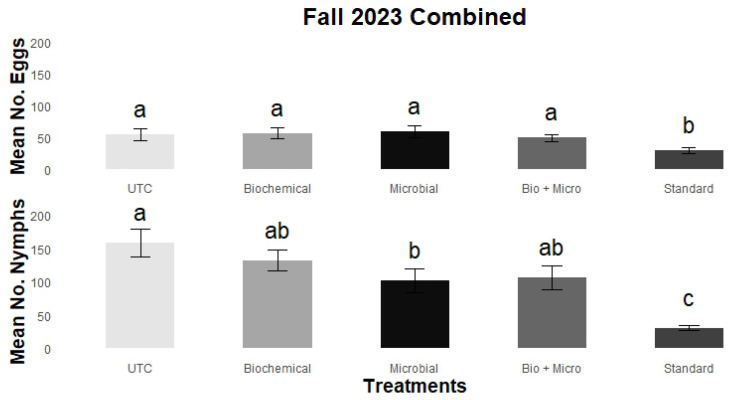
Mean (±SEM) number of eggs (**top**) and nymphs (**bottom**) combined for field trial carried out at GCREC during fall 2023. Tukey’s mean separation letter designates statistical differences within treatments (insecticide rotations). Columns with different letters are statistically different (*p* < 0.05).

The combined data presented in Figure 4 further illustrate the overall impact of insecticide rotations throughout the fall 2023 trial. The standard synthetic rotation consistently demonstrated significant reductions in both mean egg (29.2 ± 5.1) and mean nymph (30.9 ± 4.1) counts compared to all other treatments. The other insecticide rotations had mean eggs ranging from 49.3 ± 6.1 (bio + micro) to 59.7 ± 9.7 (microbial) and mean nymphs ranging from 101.9 ± 17.6 (microbial) to 158.5 ± 20.8 (UTC). Their mean egg counts were not significantly different (*p* > 0.05). However, the microbial treatment, with a mean nymph count of 101.9 ± 17.6, showed a significant reduction in mean nymph counts when compared to the UTC (158.4 ± 20.8) but did not significantly differ from the biochemical (132.0 ± 15.9) or bio + micro rotations (106.3 ± 15.9).

## 4. Discussion

The 2023 field trials at the UF GCREC were designed to compare various biopesticide rotations and a standard synthetic insecticide treatment for managing MEAM1 whiteflies. The main objective of our study was to assess how different biopesticide rotations impact *B. tabaci* management compared to synthetic insecticides. The results of both trials revealed a significant influence of rotation treatment and sample date on whitefly egg and nymph populations. Nevertheless, both trials highlighted a lack of significant interaction between rotation treatment and sample date. This may suggest that while some insecticide rotations affected pest populations, their influence was independent of the time frame over which samples were collected. The lack of significant interaction may indicate that the effectiveness of the rotations did not vary significantly over time, or it could reflect a need to further refine the timing and combination of pesticide applications for optimal pest control.

In both spring and fall trials, M-Pede was applied alone during the two initial weeks of the biochemical rotation, as well as combined with BG and PFR before samples 1 and 4 were collected, respectively. Overall, those samples resulted in egg/nymph counts that were not different from the UTC. Previous greenhouse [40] and field [42] studies had contrasting results, with M-Pede being effective in reducing both adult and immature whitefly populations. Although M-Pede was applied carefully in our study, field conditions inherently introduce more variability and challenges in ensuring thorough coverage, especially on plants with complex architectures like tomatoes. These differences could also result from environmental conditions, application methods, and plant structures that may contribute to variations observed in M-Pede performance [46]. Furthermore, M-Pede may precipitate in hard water, which could affect its efficacy in field applications [46].

SX was applied during the mid-season of the biochemical rotation and combined with BG and PFR before samples 2 and 5 were collected, respectively. This material demonstrated an inconsistent, low impact on egg and nymph counts when applied alone or combined, with no significant differences compared to the UTC. Previous studies have demonstrated that SX has potential to control *B. tabaci* in greenhouse settings [38,39]. However, our study indicates that careful considerations must be taken when deciding to use SX in commercial tomato fields. Variables such as temperature, humidity, and plant surface characteristics may affect the coverage and efficiency of mineral oils. An open environment with variable microclimates and structural complexities of tomato foliage might pose challenges for achieving thorough coverage and a maximum effectiveness of SX. This underlines the importance of refining application strategies, including timing, concentration, and coverage, to improve the performance of SX under varying field conditions.

Trilogy was applied in the later weeks of the biochemical rotation and combined with BG (samples 1 to 3) and PFR (samples 4 to 6). As mentioned above, the sixth sample was not collected during the spring trial. This material showed consistent low efficacy in managing *B. tabaci* eggs and nymphs across both seasons, being statistically similar to the UTC. The primary active ingredient in Trilogy is azadirachtin, a triterpenoid derived from the neem tree *Azadirachta indica* A. Juss (Meliaceae), known for its minimal toxicity to humans and lesser harm to non-target organisms compared to other botanical biopesticides [56]. Despite these benefits, the major challenge with neem-based products lies in their rapid photodegradation, particularly due to UV radiation, when applied as foliar treatments [47]. This vulnerability to external factors such as UV light is exacerbated when neem is deposited on the leaf surface through foliar application [47]. Interestingly, neem has shown potential as an oviposition deterrent, with fewer eggs laid on treated leaves compared to untreated controls, indicating its role in disrupting the reproductive cycle of whiteflies [56]. However, our study suggests that while neem alone may offer limited control against *B. tabaci*, its efficacy could potentially be enhanced when combined with a biological control agent such as *B. bassiana*. This combination has been shown to increase *B. tabaci* mortality [57]. Similarly, we found a 44.6% reduction in nymph counts in our third sample during the fall of 2023, which was collected one week after BG (*B. bassiana*) was applied in combination with Trilogy. However, this reduction was not significantly different compared to the UTC nor consistent across both spring and fall trials.

BG, a key component of the microbial rotation, showed promising results in the spring trial by significantly reducing egg counts by 90.5% in the first sample in comparison to the UTC. However, its effectiveness tended to decrease in later samples during the spring, with marginal reductions of 26.7 to 33.1% in egg counts. Overall, BG contributed to a significant reduction in egg counts when compared to the UTC across the spring trial. In the fall trial, BG foliar applications did not reduce egg counts in any of the analyzed samples. Our results showed that the BG impact on nymphs was non-significant but notable, reducing nymphs by 34.4% on average compared to the UTC. Due to its slow-acting control on *B. tabaci*, *B. bassiana* may not prevent the primary transmission of TYLCV by viruliferous adults migrating into the field given that TYLCV can be transmitted within a few minutes of feeding [41,58]. However, *B. bassiana* and other entomopathogenic fungi of equivalent mode of action could decrease the secondary transmission of TYLCV by controlling *B. tabaci* immatures derived from migratory populations [41]. These variations in performance might be attributed to the influence of environmental conditions on fungal infections. The infection rates of BG significantly depend on the duration of high-humidity exposure, with about 50% infection achievable even in low humidity, and ~47 h of high humidity required for >90% infection by *B. bassiana* emulsion [59]. Such performance may highlight its limitations as a standalone treatment and underscores the importance of incorporating BG into a multifaceted approach, including higher-humidity conditions.

The temporary use of cages, which can potentially increase humidity, should be evaluated when applying *B. bassiana*-based insecticides in future studies. When combined with the biochemical insecticides mentioned above, BG tended to consistently reduce egg and nymph counts to a marginal degree across both trials. This reduction, however, was not statistically significant compared to the UTC. The most notable reduction occurred when BG was combined with SX (sample 2) in both trials, with nymph counts being reduced by 41.2% during the spring and by 73.5% during the fall. This aligns with a previous report that *B. bassiana* treatments significantly impacted *B. tabaci* populations, reducing egg numbers by up to 65% and nymph numbers by up to 58% compared to untreated plants [45]. In our study, we used the highest concentrations of these materials according to each product label. Future studies are warranted to investigate if different rates of BG plus SX could be more effective in providing *B. tabaci* control.

In the spring trial, PFR provided a marginal reduction in egg counts by up to 57.1% in the fifth sample compared to the UTC, and up to a 36.6% decrease in nymph counts in the same sample. The overall impact of PFR as part of the microbial rotation along with BG was significantly greater than UTC for eggs but statistically non-significant for nymphs during the spring trial. In the fall trial, PFR did not significantly decrease egg counts in any of the analyzed samples, whereas its overall impact as part of the microbial rotation significantly decreased nymph counts compared to the UTC. Furthermore, combining different biochemical insecticides with PFR was not effective across both seasons. A previous study found that repeated applications of PFR as a foliar spray were more effective in managing the invasion of the Ficus whitefly, *Singhiella simplex* (Singh), compared to the untreated control, suggesting the potential for its effective use against similar pests [60]. Environmental conditions may significantly influence the effectiveness of fungal biopesticides such as *C. javanica*. The optimum virulence of *C. javanica* occurs at 25 °C, with reduced virulence at higher temperatures (>30 °C) [44]. Additionally, a brief UV exposure of 5 to 10 min can cause significant mortality to some strains of *C. javanica* [44]. Therefore, reducing UV exposure during and after the application of *C. javanica* is essential for successful fungal development. Techniques might include applying PFR during times of low UV intensity, incorporating UV-protective cages, or developing UV-resistant strains of *C. javanica*.

Dinotefuran was part of the standard rotation and demonstrated a marginal reduction of 74.1% in egg counts and a significant reduction of 75.9% in nymph counts during the spring trial. We also observed significant reductions of 89.7% and 88.6% in nymph counts during the spring trial. In addition, dinotefuran demonstrated a significant reduction of 76.6% in egg counts and no reduction in nymph counts during the fall trial. We also observed significant reductions of 92.6% and 86.9% on nymph counts during the fall trial. The soil characteristics of the GCREC experimental site, particularly being a Myakka fine sand with a 98% sand content and low silt (1%) and clay (1%) [61], may lead to increased drainage and reduced water retention. As a result, water-soluble insecticides like dinotefuran can quickly leach beyond the root zone, which might reduce their availability for plant uptake. However, the overall effectiveness of dinotefuran observed in our trials highlights its strength as a systemic insecticide, establishing a solid base for pest control within the treatment rotation.

Cyantraniliprole, used in the mid-season of the standard rotation, demonstrated marginal reductions in egg and nymph counts during both spring and fall trials, except for a significant reduction in egg counts observed in sample 3 during the fall trial. While the decrease in egg and nymph counts was not statistically significant, the efficacy of cyantraniliprole was notable, reducing egg counts by 77.6% and nymph counts by 79.6% in the spring trial, and by 58.9% and 66.7%, respectively, in the fall trial. This performance aligns with studies that have reported cyantraniliprole’s intermediate to high effectiveness against MEAM1 [7,15,18,62,63]. However, cyantraniliprole may not be sufficient to completely manage MEAM1 populations and TYLCV transmission under higher pest densities [18]. The rise in resistance to cyantraniliprole is a growing concern in southern U.S. A recent study observed low to moderate levels of MEAM1 resistance to cyantraniliprole in Florida, particularly in Hillsborough County where our trials were conducted [23]. This emerging resistance is further supported by field scout data from Georgia, indicating a change in the efficacy response to cyantraniliprole and suggesting a potential rise in resistance to this insecticide [22]. Therefore, while cyantraniliprole has shown promise in managing *B. tabaci* and other pests, the evolving resistance patterns underline the need for continuous monitoring and potentially integrating it with other pest management strategies to maintain its effectiveness.

Buprofezin, used in the mid-season of the standard rotation, demonstrated marginal reductions in egg and nymph counts during both spring and fall trials. While the decrease in egg and nymph counts was not statistically significant, the efficacy of buprofezin was notable, reducing egg counts by 67.0% and nymph counts by 77.7% in the spring trial, and by 47.2% and 60.6%, respectively, in the fall trial. The high effectiveness of buprofezin in suppressing MEAM1 nymphs has been reported in Florida [8].

Pyriproxyfen, used in the later season of the standard rotation, demonstrated marginal reductions in egg counts and significant reductions in nymph counts during the fall trial. The reduction in egg counts was not significant, yet pyriproxyfen effectively reduced egg counts by 39.0% and significantly reduced nymph counts by 74.6% in the fall trial. In Florida, a high efficacy of pyriproxyfen in managing MEAM1 nymphs has been demonstrated [8]. In contrast, a more recent study conducted in Georgia reported a lower efficacy of pyriproxyfen against MEAM1 populations [22]. However, a low to high resistance to pyriproxyfen has been observed in the MEAM1 population globally [26,27]. This global trend of resistance to buprofezin and pyriproxyfen emphasizes the necessity for the continuous monitoring of resistance development of MEAM1 populations to these insecticides in Florida. Our results reveal the importance of exploring alternative or complementary management strategies, such as the rotation of insecticides with different modes of action, to maintain the efficacy of buprofezin and pyriproxyfen in *B. tabaci* management programs.

Afidopyropen, included in the later weeks of the standard rotation, demonstrated a significant reduction of 89.4% in egg counts and a marginal reduction of 84.1% in nymph counts during the spring trial. In contrast, we observed a marginal reduction of 48.7% in egg counts and a significant reduction of 78.0% in nymph counts during the fall trial. The efficacy of afidopyropen against MEAM1 populations has not been extensively evaluated in Florida. However, a recent report indicates low to moderate resistance levels in two MEAM1 populations [23], including a population from Hillsborough County, the same County where we conducted our trials.

The consolidated findings from both the spring and fall 2023 trials highlight the complex dynamics of insecticide rotations and their impact on *B. tabaci* management. In the spring, the standard synthetic rotation was highly effective, significantly reducing both egg and nymph counts by 78.6% and 83.5%, respectively, compared to the UTC. Interestingly, the standard rotation’s impact on egg counts was not significantly different from the microbial rotation, which provided a reduction of 49.2% in eggs counts compared to the UTC. Although the microbial rotation did not provide a statistically significant reduction in nymph counts compared to the UTC, this rotation reduced the number of nymphs by 34.0%. In addition, the standard rotation was significantly more effective than the biochemical and bio + micro rotations, which had similar egg (95.8% and 100%, respectively) and nymph (95.4% and 83.2%, respectively) counts in comparison to the UTC. This pattern was mirrored in the fall, where the standard synthetic rotation again demonstrated a substantial reduction in both mean egg (46.8%) and nymph (80.5%) counts compared to the UTC, reinforcing its consistent efficacy across seasons.

The microbial rotation, which was effective to some extent in the spring, exhibited a significant reduction in nymph (35.7%) counts during the fall when compared to the UTC. This reduction in nymph counts, however, was not significantly different from the reduction provided by the biochemical (16.7%) and bio + micro rotations (32.9%) when compared to the UTC. For egg counts, the biochemical (>100%), microbial (>100%), and bio + micro (89.8%) rotations were statistically similar to the UTC. These observations highlight the overall efficacy of the standard synthetic rotation in suppressing whitefly populations and underscore the potential of microbial treatments in integrated pest management. These results align with the established understanding that synthetic insecticides are generally more effective in providing pest control compared to biopesticides [43,64]. Furthermore, we did not observe significant synergistic or antagonistic effects when combining microbial insecticides containing *B. bassiana* or *C. javanica* with the insecticidal soap (M-Pede) or mineral oils tested (SX and Trilogy). Our results indicated a neutral effect when combining these insecticides under the climatic conditions of our study. Previous studies that evaluated the compatibility between *B. bassiana* and *C. javanica* with mineral oils reported neutral, antagonistic, or synergistic effects among these mixtures, which are likely to be attributed to different formulations of the non-microbial insecticide [65,66,67]. Overall, our findings suggest potential for microbial biopesticides in IPM strategies. Nonetheless, the observation that microbial rotations occasionally mirrored the performance of other non-synthetic insecticides and UTC rotations throughout our trials indicates a need for optimizing the application of microbial biopesticides to achieve their full potential. Future studies are warranted to investigate the potential of integrating microbial insecticides as part of a standard rotation of modes of action to manage MEAM1 in Florida vegetable fields.

## 5. Conclusions

In summary, the present study demonstrated the influence of insecticide rotations on MEAM1 populations, with synthetic treatments showing consistent efficacy across seasons and microbial insecticide rotations offering potential as part of IPM programs. The results of this research highlight the further necessity of exploring the rotation of insecticides with different modes of action and integrating non-chemical control measures. Our findings suggest that the simple combination of different types of biopesticides may not inherently enhance control efficacy and may require additional strategies to optimize their use. The fluctuating performance of biopesticides across different trials illustrates the complex interplay of factors such as environmental conditions and application timing. Future research should focus on exploring the integration of biopesticides into standard rotation programs assessing the compatibility of microbials in a standard rotation, and optimizing application technologies. This study not only contributes to the current understanding of MEAM1 management but provides a foundation for further research to optimize sustainable control strategies in agricultural settings.

## Figures and Tables

**Table 1:** Treatments, material applications, and sampling timetable post transplantation to evaluate the effectiveness of rotating biopesticides and synthetic insecticides in managing MEAM1.

Treatment	Rotation	Material	Week Applied ^1^	Sample No. ^2^
**1**	Control	No material applied	-	
**2**	Biochemical Insecticides	M-Pede	3–4	1–2
		SuffOil-X	5–6	3–4
		Trilogy^®^	7–8	5–6
**3**	Microbial Insecticides	BotaniGardES	3–5	1–3
		PFR-97 20WDG	6–8	4–6
**4**	Treatments 2 and 3 combined (bio + micro)	BotaniGardES + M-Pede	3	1
		BotaniGardES + SuffOil-X	4	2
		BotaniGardES + Trilogy^®^	5	3
		PFR-97 20WDG + M-Pede	6	4
		PFR-97 20WDG + SuffOil-X	7	5
		PFR-97 20WDG + Trilogy^®^	8	6
**5**	Standard	Dinotefuran	at-plant	N/A
		Dinotefuran	3	1–2
		Cyantraniliprole	5	3
		Buprofezin	6	4
		Afidopyropen	7	5
		Pyriproxifen	8	6

^1^ Application time expressed in weeks after transplanting tomato to the field. ^2^ Sample number related to the week (s) post transplanting in which tomato terminal leaflets were collected.

**Table 2:** Statistical parameters comparing the effect of rotation treatment, sample date, and the interaction between these factors on *B. tabaci* eggs and nymphs for field trials carried out at GCREC during the spring and fall of 2023.

Trial	Factor	Eggs	Nymphs
**2023 Spring**	Rotation	F_4,75_ = 10.11; *p* ≤ 0.0001	F_4,75_ = 21.63; *p* ≤ 0.0001
	Sample date	F_4,75_ = 16.44; *p* ≤ 0.0001	F_4,75_ = 24.94; *p* ≤ 0.0001
	Rotation * Sample date	F_16,75_ = 1.32; *p* = 0.2099	F_16,75_ = 1.33; *p* = 0.2022
**2023 Fall**	Rotation	F_4,87_ = 4.49; *p* = 0.0024	F_4,87_ = 21.47; *p* < 0.0001
	Sample date	F_5,87_ = 0.54; *p* = 0.7487	F_5,87_ = 9.04; *p* < 0.0001
	Rotation * Sample date	F_20,87_ = 0.96; *p* = 0.5151	F_20,87_ = 1.31; *p* = 0.1831

## Data Availability

Dataset available on request from the authors.
